# Students’ multimodal knowledge sharing in school: Spatial repertoires and semiotic assemblages

**DOI:** 10.1007/s10639-021-10837-0

**Published:** 2021-12-15

**Authors:** Linnea Stenliden, Jörgen Nissen

**Affiliations:** Department of Behavioural Sciences and Learning (IBL), Linköping University, Campus Norrköping, SE 601 74 Norrköping, Sweden

**Keywords:** bodily expressions, knowledge sharing, multimodality, school, semiotic assemblage, spatial repertoires, visual analytics, verbal expressions

## Abstract

In a world ‘flooded’ with data, students in school need adequate tools as Visual Analytics (VA), that easily process mass data, give support in drawing advanced conclusions and help to make informed predictions in relation to societal circumstances. Methods for how the students’ insights may be reformulated and presented in ‘appropriate’ modes are required as well. Therefore, the aim in this study is to analyse elementary school students’ practices of communicating visual discoveries, their insights, as the final stage in the knowledge-building process with an VA-application for interactive data visualization. A design-based intervention study is conducted in one social science classroom to explore modes for students presentation of insights, constructed from the interactive data visualizations. Video captures are used to document 30 students’ multifaceted presentations. The analyses are based on concepts from Pennycook (2018) and Deleuze and Guattari (1987). To account for how different modes interact, when students present their findings, one significant empirical sequence is described in detail. The emerging communicative dimensions (visual-, bodily- and verbal-) are embedded within broad spatial repertoires distributing flexible semiotic assemblages. These assemblages provide an incentive for the possibilities of teachers’ assessments of their students’ knowledge outcomes.

## Students’ modes for knowledge sharing – attention to emerging changes due to powerful technologies in school

New demands are placed on the requirements for how students in school both need to be trained to use appropriate tools in order to develop their skills to interpret/analyse large volumes of data and to be encouraged to complete such problem-solving processes in ways that facilitate adequate ways to present their knowledge (Bodén & Stenliden, 2019; Nissen & Stenliden, 2020). The demands emerge due to the volume of data in the world that has exploded in recent years. The growth will continue at an incredible rate (Marr, 2020) and is even higher than previously expected caused by the increased challenges due to the COVID-19 pandemic, as more people worked and learned from home (Holst, 2021). Already there is a conspicuous divergence between the huge amount of information produced, collected, retained and organized and the human capacity to make sense of and learn from it (Stenliden, Nissen & Bodén, 2017, p 185).

Visual analytics (VA) is a technology developed to provide data visualization tools that help people to understand the significance of large volumes of data by placing them in multimodal (visual) contexts (Andrienko, 2013). Patterns, trends and correlations that might go undetected in text-based documents can be exposed and easier be recognized with VA tools such as Gapminder (Rosling, Ronnlund, & Rosling, 2007) Statistics eXplorer (Lundblad, 2013), Tableau (Hoelscher & Mortimer, 2018) or Qlick View (Serbanescu, 2018). The VA applications have become valuable to society due to their power to support human understanding of circumstances in society and prevalent in many areas as they contribute to various kinds of problem-solving (Andrienko & Andrienko, 2020).

If students in school are introduced to these powerful multimodal (artificial intelligence (AI)) technologies, this helps them to collaboratively manage analysis of large amounts of data and develop insights (Stenliden, 2014, 2015). Empirical evidence shows that intelligence systems involving students in a partnership improves their knowledge outcomes (Roll & Wylie, 2016; Luckin, 2018). At the same time, the ways in which the students’ visual discoveries (knowledge) can be shared and communicated with others (as well as assessed) are also influenced. The students often end up with problems concerning ‘the reformation’ of insights from multimodal knowledge processes (Hashemi, 2019; Stenliden, 2014; Åkerfeldt, 2014).

One major origin of these problems seems to be that, traditionally, students’ knowledge must be recognizable and measurable in school. Thus, the final assignment for students’ is usually to produce a written text, as ‘proof’ of having achieved knowledge (Åkerfeldt, 2014). Regrettably, this rather ‘static’ mode often seems to narrow the possibilities to transfer and demonstrate knowledge gained with multimodal technology. Studies have shown that, even though students rather easily detect patterns between indicators, identify relations and draw conclusions that, without technological support, would have been impossible for them to harvest (Lundblad, 2013), they do have difficulties in representing their insights as they become more complex, with support from tools such as data visualizations (Stenliden, 2014). Stenliden (2015) showed in her study how the students ended up in various ‘problem spaces’ as they were not able to efficiently document, represent and present the complex circumstances only by a written text mode. As suggested by many researchers (cf. Baldwin, 2015; Baldwin, 2016; Bearne, 2009; Cope & Kalantzis, 2000; Purdy, 2014), when working with multimodal technologies in school, it is important not only to thoroughly develop students’ ability to understand what is seen, interpret what is experienced, analyse what they have been exposed to and evaluate and draw conclusions based on criteria that support critical thinking. But flexible methods for how the students’ insights may be reformulated and presented in an ‘appropriate’ mode must also be developed. Students would be better off if they, as a final assignment are encouraged to produce ‘proof’ of achived knowledge in multimodal ways that may promote them better (Baldwin, 2016).

In other words, it is essential to investigate various ways in which students can share insights and communicate knowledge in ways that allow demonstrations of complex patterns and conclusions. Therefore, this study explores modes that may be flexible and modifiable in providing assistance for elementary school students presentations of their insights, constructed from interactive data visualizations in relation to social-science content. More specifically, the aim is to analyse students’ practices of communicating visual discoveries, their insights, as a final stage in the knowledge-building process. The following research question is addressed:

- How are various modes (visual, bodily and verbal) brought together and function when students findings from visualized statistics are presented in a social science classroom?

The study builds on a design-based research project where a specific intervention is conducted. The particular VA, Statistics eXplorer^[^1^]^ (Lundblad, 2013), is applied by two teachers who experiment with novel didactic designs in two social science classrooms with their 13–14-year-old students. The study contributes to awareness of how powerful digital media, when introduced in school, also requires multimodal knowledge sharing and discusses the growing importance of understanding the composition of such presentations.

## Theory: Distributed communication as semiotic assemblages

Thinking about practices of communication and people is often an enterprise that is understood as located within humans, but at the same time Pennycook (2018) highlights that sensory (bodily, visual or oral) experiences are not separated from materiality, spatiality, artifacts, entities, etc. Consequently, when students (and others) communicate insights, many different factors come into play as a complex act of resemiotization (Iedema, 2003) or, as Pennycook (2010) puts it, a relocalization emerges. Therefore:

this is not only a process of remaking meaning in different contexts or making inscriptions of different meanings onto different surfaces, but also of redistribution of meaning in physical space, a reorientation of meaning in relation to the human body and the physical surroundings. (Pennycook, 2018, p. 40)*.*

This is the case, with Mitchell’s (2003) suggestion that communication should be seen without a clear distinction between internal cognitive processes and external material, mechanical and computational ones. As humans we perceive, act, discover, learn, and know through such processes and the extended bodies that we construct and reconstruct for ourselves. They play semiotic roles, they are parts of communicative routines constructing spatial repertoires in distributed communication (Pennycook, 2018). The notion of repertoire can be understood as an emergent and interactant affordance of a ‘communicational space’, rather than an individual and communal capacity. Observing interactions between linguistic and spatial resources, as in a classroom with all its flows of activities, linguistic and material resources, and people, it becomes evident that the notion of repertoire is better understood as spatial and distributed, not tied only to individuals or communities. Therefore, a range of semiotic resources are distributed within and beyond spaces where people, artefacts and practices interact, creating networks. Looking across classrooms, and other sites, it becomes obvious that communication is embedded within broad spatial repertoires. Approaching this performative mimesis in terms of transitory semiotic assemblages (Deleuze & Guattari, 1987; Pennycook, 2017; Hua, Otsuji & Pennycook, 2017) enables a study of communication as an inter-bodily, multi-sensory coordination in real-time where communication occurs by attuning (human and non-human) bodies (Pennycook, 2018, p. 105–106).

Pennycook (2021) argues that a focus on semiotic assemblages reconfigures what counts as language and how social and spatial worlds interact. An understanding of semiotic assemblages opens for an appreciation of how different ad hoc groupings of diverse elements, people, semiotic resources, vibrant materials of all sorts come together in particular moments. To quote Appadurai (2015, p. 221) semiotic assemblages “are temporary arrangements of many kinds of monads, actants, molecules and other dynamic ‘dividuals’ in an endless, non-hierarchical array of shifting associations of varying degrees of durability”. Thus, the concept serves to grasp the entangled, temporal multiplicity of communication in a classroom where biological/cognitive, social/material and verbal/bodily entities, the many forms of semiotics or sign processes interacts (Pennycook, 2017, p. 269). The notion enables a way of thinking not so much of on language use in specific contexts, but rather how the spatial gathering of linguistic resources and other material elements e.g., things, visuals, bodies, words and places connect and co-operate in specific moments in time (Martín Bylund & Stenliden, 2020; Pennycook, 2021).

When studying the ways in which things are brought together and function during practices of communicating a message of visual discoveries, we consider spatial repertoire to be an apt analytical tool (Pennycook, 2018). It offers a way of thinking about communication as distributed in socio-material interaction. We also adopt semiotic assemblage for what could be deemed an understanding of communication as corporeal and affective (Deleuze & Guattari 1987) as we try to observe the interactions between linguistics, visual properties, bodies, and other spatial resources that may emerge in the studied communicational space of the classroom. To further consider how various resources or modes are generally understood to intersect, the following section introduces previous research focusing on practices of communication when insights or messages of visual discoveries are to be expressed.

## Previous research: Expressing insights

Communicating through visual messages is compound and therefore the essential characteristics cannot be defined only in terms of perceptions of visualization, print, and writing (Tufte, 1997). There is also a plethora of concepts representing different dimensions of communicative practices to define the symbolic representation of ‘things’ – facts – insights – knowledge and the process of sharing or communicating a message (Fahmy, Bock, & Wayne, 2014; Horn, 1998; Pettersson & Avgerinou, 2016; Sankey, Birch, & Gardiner, 2011). Latour (1990, p. 36) describes how images and inscriptions function in rhetorical or polemical dialogues:

You doubt of what I say? I’ll show you. And, without moving more than a few inches, I unfold in front of your eyes figures, diagrams, plates, texts, silhouettes, and then and there present ‘things’ that are far away and with which some sort of two-way connection has now been established. I do not think the importance of this simple mechanism can be overestimated*.*

Latour (1990) demonstrates what happens when a few people in the same room *talk* to one another *together with* two-dimensional pictures; these visualizations, although some are powerful, are all there is to see of the ‘things’ about which they talk, but they forcefully enhance the understanding. In other words, in this world of media and visualizations that we are used to, it is easy to understand the need to use references, tables, graphs, diagrams, photographs, peaks, spots, bands, etc. to understand and explain something. Of course, not all explanations in terms of inscription or images are equally convincing, only those that help us to understand how the mobilization and mustering of new resources is achieved (Latour, 1990).

Means, strategies, tools, and methods to make knowledge visible through visual communication (Griffin, 2008) are developed in the domain of knowledge visualization (KV) (Eppler, 2013). The focus is to improve the processes through which ‘things’ (knowledge) can be identified, assessed, shared, discussed, applied, and generally managed (Davenport, 2011). Tversky and Suwa (2009) highlight that KV is a creative process specifically involving sketches with combinations of images, words in text, arrows, lines etc. They argue that creating such visuals helps to externalize ideas, make them more permanent and facilitate comprehension and inference. The visuals are artifacts that allow community participation and checks for completeness and consistency. According to Eppler and Burkhard (2007), if one wishes to communicate convincingly supported by visuals, the information, knowledge, opinion, etc., that is gathered and displayed must be presentable all at once to the receiver of the message. The ‘things’ have to be acceptable, readable, and combinable in a logical manner (Latour, 1990), for example, giving an overview of details or presenting a top-to-bottom process in a problem analysis (Tufte, 1997). In this scenario, visual discovery is understood as the pursuit of captured, novel insights to take on a different form, as they are generated out of the analysis and mapping of mass data and then visualized via individual and/or collective views, opinions, and analyses (Ryan, 2016). This is to link ‘things’ in various ways in order to facilitate acceptable discoveries from shared insights (Suthers, 2001). Likewise, in Bertschi’s et al. (2013) opinion, re-presenting insights is a non-linear process. He argues that any step in the process of gathering, organizing, and designing can link to any other step, in any number of iterations, until a ‘final’ representation is created. When trying to express visual discoveries through visual communication, there are always choices to be made due to visual narrative tactics and design space considerations (Segel & Heer, 2010). Eppler (2013) introduces the concept visual playfulness to emphasize the importance of employing a playful exploratory and creative approach to putting elements together, so they become readable as transitional objects, that enable and contribute to an open, logical dialogue around the visualization. Thus, the visual should also provide a way to reframe issues and persuade participants into different interpretations, thus generating new insights. Eden and Ackermann (2006) argue that this form of playful interaction wastes less energy in impression management and that the knowledge exchange may be more immersive than it would otherwise be.

Kress and Van Leeuwen (2006, p. 177) propose that the representational and interactive composition and meaning of an image relates to three interconnected aspects. They highlight how (1) the placement of elements, for example the various ‘zones’ of the image, left/right, top/bottom, centre/margin create information value/quality, (2) the salience of elements, for instance positioning items in the foreground or background, or using size, contrasts in tonal value or colour, differences in sharpness, etc. attract the viewer’s attention (3) the presence or absence of framing devices such as lines divide, connect or disconnect elements in the image and manifest if something belongs there, belongs together or does not belong together at all, etc. According to Tufte (1997), when many visual events are selected and combined, as discussed here, a visual confection is produced. A visual “confection illustrates an argument, presents and enforces visual comparisons, combines the real and the imagined, and tells yet another story” (p. 121). Through visual guidance, images also act as signposts to the order in which the visualization should be ‘read’ and discussed in a combinable manner (Eppler, 2013). To sum up, a KV should amount to: a communicable image, consisting of various visual notations, that is interactively annotated in a playful, creative, yet systematic manner that explains, leads to understandings and new discoveries while remaining flexible to incorporating possible future revisions and insights.

For students in school, it is often not enough to express their insight through visual communication, also dialogue and a reasoning practice are required to share their knowledge to their teacher and fellow students (Hunt et al., 2014; Nissen & Stenliden, 2020). The basis for such public speaking is often considered as the combination of students’ knowledge, skills, and attitudes to communicate/speak in public (De Grez, et al., 2009; Van Ginkel et al., 2017; Herbein, et al., 2018). Herbein et al. (2018) deepen this conceptualization of speech performance and explain it by four micro-level skills (1) nonverbal behaviour–visual impression (including eye contact, gestures, mimicking, proxemics: i.e., spatial behaviour, usage of notes), (2) nonverbal behaviour–auditory impression (including accentuation, articulation, breaks, intonation, volume, pitch, speech fluency, speech rate, speech i.e., respiration used when speaking, voice), (3) language usage (including activation of the listener, linguistic expression, personal address, usage of rhetorical devices), and (4) organization (including amount of information, intention of communication, length of speech, length of introduction). Schreiber, Paul, and Shibley (2012) argue that the two primary responsibilities of a competent ‘speaker’ are putting together the content of the message and delivering that content well.

**Fig. 1:**
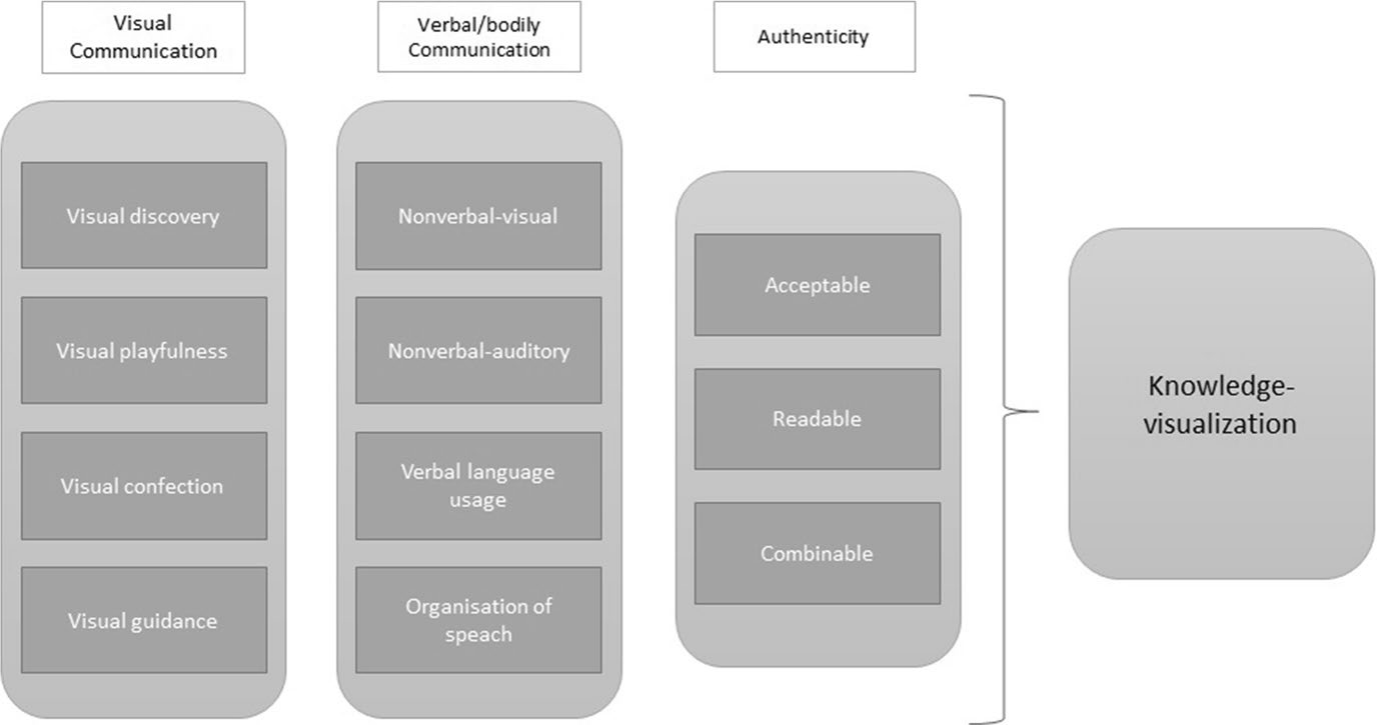
Dimensions of visual communication and dimensions of verbal/bodily communication and how these aspects, if they are perceived as acceptable, readable, and combinable, may establish authenticity.

In the literature, these dimensions of communication (visual, verbal, and bodily) are often viewed quite separately, but all have inherent qualities which constitute parts of the processes when creating and sharing knowledge with others (Fig. 1). For example, in a study of presentation skills training together with technology by Batrinca et al. (2013), only presentation skills and multimodal information about the alignment of body movement, gestures, and facial features was evaluated. Joe et al. (2015) state that research is needed to determine how technology can be used to support public speaking. Such performance was evaluated by Feng et al. (2019), based on content-related (e.g., speech organization, word choice) and delivery-related (e.g., vocal expression, nonverbal behaviours) dimensions. Content-related dimensions were scored based on speech transcripts, while delivery dimensions were scored using video techniques. This is an attempt to achieve a broader scope; still, there are few examples of merging visual and verbal and bodily dimensions in analyses of communication.

To recall, Kress and Van Leeuwen (2006) argue that, even when we can express what seem to be the same meanings in either image-form or writing or speech, they will be realized differently. How something is expressed, verbally/bodily and/or visually, makes a difference (Kress & van Leeuwen, 2006). For instance, something that is expressed in verbal communication through the choice of different word classes and clause structures may be articulated in visual communication through the choice between different uses of colour or different compositional structures, and this will affect meaning (Griffin, 2008). So, how this multifaceted process, including social cognitive processes, external material, mechanical, and computational ones, as well as the interplay of visual, verbal, and bodily communication (Pennycook, 2018), is explored in this study will be outlined in the next section.

## Method

The study’s empirical data consists of presentations made in elementary school of individual students (n = 30), grade eight, in which they communicated visual discoveries, their insights. Each student gave their presentation to a small group, 6–7 other students, and the teacher as a final stage in the knowledge-building process. These presentations served as examinations of the students’ previous work. They were video recorded by the researcher who were also present during the presentations.

The presentations, i.e the examinations, are part of an extensive intervention study, designed by teachers and researchers who worked together within the framework of design-based research methodology (DBR) to develop, implement, and study a specific classroom intervention through several stages (Anderson & Shattuck, 2012; Easterday, Rees Lewis, & Gerber, 2016). The included data in this study, focuses on the examinations lessons where the students’ insights are presented. In total the intervention was followed during 24 lessons (cf. Bodén & Stenliden, 2019; Nissen & Stenliden, 2020; Stenliden, Nissen & Bodén, 2017; Stenliden, Bodén & Nissen, 2019). In order to place the examining presentations in an understandable context, we describe the intervention in the following.

### A comprehensive intervention

The purpose of the intervention was to conduct a study of teaching where students interact with a Visual Analytics applications, in this case Statistics eXplorer (cf. Stenliden et al., 2017). The teaching included didactic design that encouraged students to, as a “final-product” of the work with VA, share insights and in their presentations in different ways illustrate complex patterns and conclusions about societal circumstances.

The VA-application used is based on information and geographical visualization and uses official statistics^[^2^]^ (Lundblad, 2013). The pedagogical intervention was built by two rounds, were adjustments and refinements of the intervention were made to the second one. The data presented in this study is from round 2.

### The setup of the intervention (round 1 and 2)

**Fig. 2:**
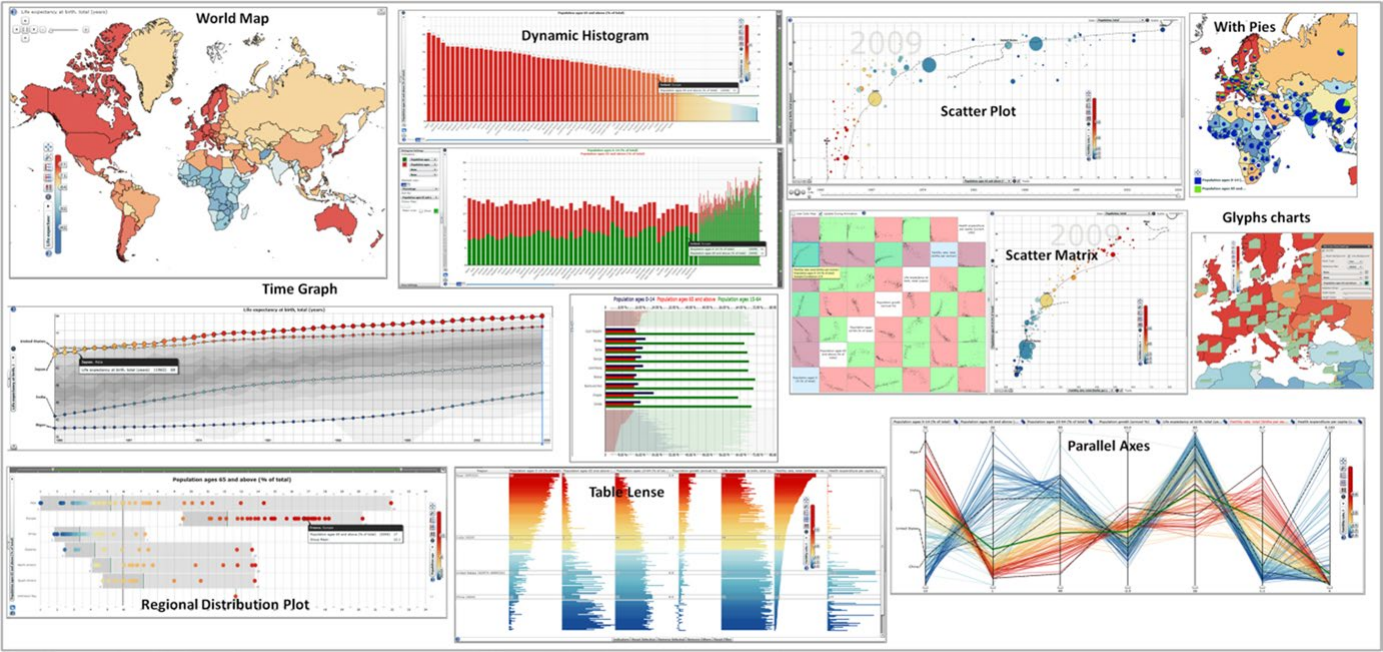
Data visualization – toolkit and interactive features, represented by layered choropleth map, composite time-linked histogram, time graph, scatter-plot view, parallel axes plot, etc.

In collaboration, the researchers and the teachers sketched a plan with workable solutions that allowed the participating teachers to design lessons plans according to the purpose of the intervention, se above. Each teacher was obliged to include the Statistics eXplorer and plan for lectures and students’ assignments according to content in social science curricula. The lessons should offer opportunities for the students to work with the VA and thereby handle large amounts of multimodal information which was supposed to contribute to their knowledge construction through ‘relevant’ facts. For the teachers this involved selecting appropriate statistics regarding the educational goals relevant to the chosen educational topics and appropriate visualizations for the content – e.g. the world map (chorophlet map), scatter diagrams, bar charts, dynamic histograms, time graphs, flowcharts, etc. (Fig. 2) and to produce visualized interactive stories.

**Fig. 3:**
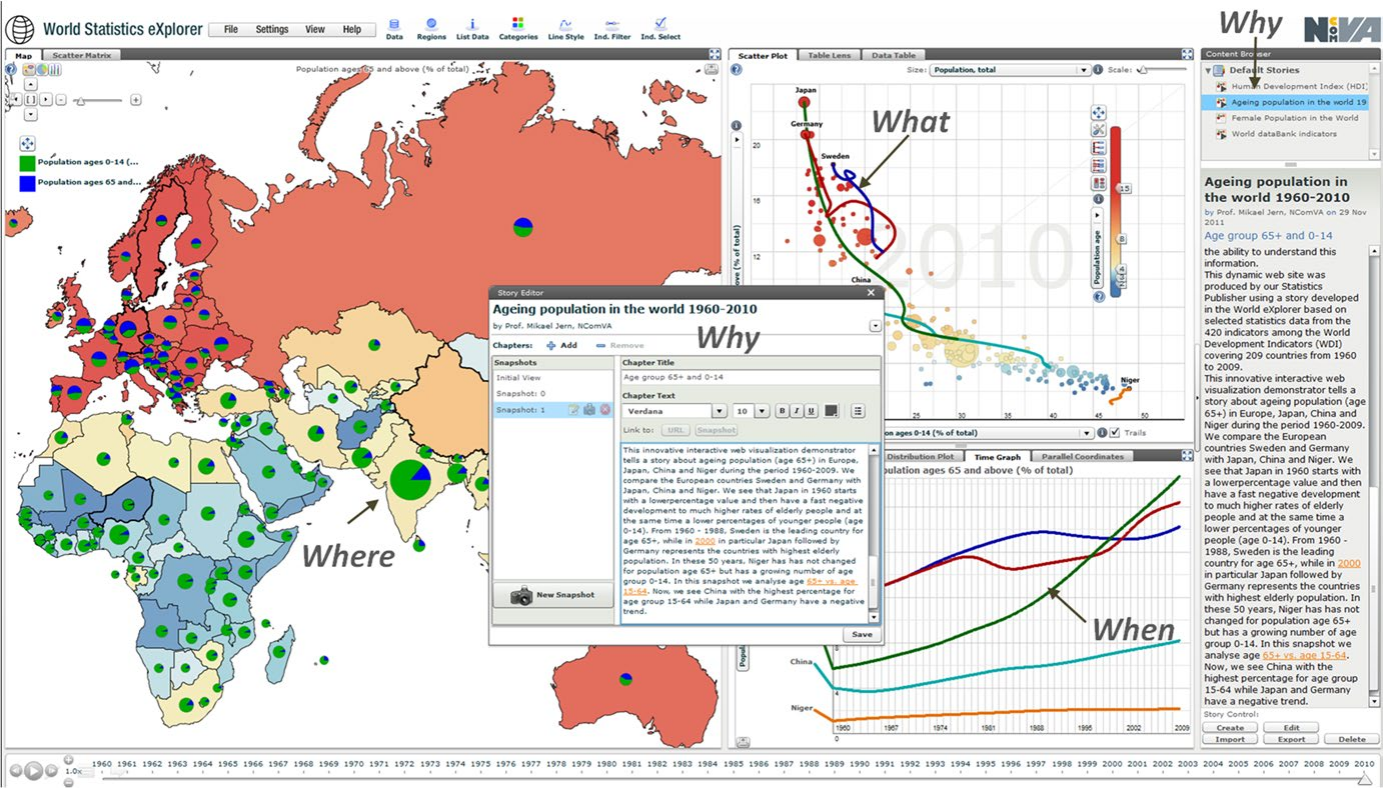
An interactive story builds on visualized statistics and text, by the combination it is possible to focus/explain why? what? where? and when? in relation to specific content.

By publishing the stories on a blog or website they became “Vislets” which is a travesty of booklet and can be viewed as a small interactive visual story where the content is selected, arranged and adjusted in relation to the viewer/reader (Fig. 3).

In the first round of the intervention there were few teacher actions that emerge in the classrooms, in relation to instructions of novel ways for the students to present knowledge, even though the teachers had included that kind of actions in their lesson plans. Hence, due to this more thorough work was done in a second round to accomplish an emphasize in the teaching that encouraged and instructed the students how to communicate messages about their visual discoveries as the final stage in the knowledge-building process.

### The teaching material and lesson plans

In round 2, the teachers collaboratively produced teaching material and the lesson plans. As these teachers, in round 2 were familiar to Statistics eXplorer, they knew how to use the applications innovative methods for data visualization. This time they transformed the content into different visuals and produced the visual interactive stories and published them as Vislets. In these, they included written text associated with the visualizations to explain specific content and created hyperlinks to zoom in on specific phenomena they wanted to visually highlight in their stories, or link to associative, descriptive text on a relevant webpage on the internet. The teachers created two Vislets together, on the theme “World Economy and Population” (GDP 1 and GDP 2). These built on four parts that contained various visual representations, with explanations in a ‘text box’. Together, these allowed a focus on the questions: what? where? when? and why? (Fig. 3). In the text box, the students’ assignments were also presented.

The teachers planned for and conducted five lessons where the students were supposed to interact with the content of the Vislets in relation to the different assignments and to prepare an oral presentation, using visualizations and ordinary text to ‘report’ their gained knowledge and conclusions based on the questions. At a sixth occasion the students were examined by their teacher through the presentation of their ‘reports’. These presentations (n = 30) are, as mentioned, the empirical data for this study.

### Classroom context

During the five lessons, the students mainly worked individually on the assignments, with help from the teacher, the two prepared Vislets in Statistics eXplorer, a written instruction document, and a digital presentation program (to create a basis for the oral presentation). The written instructions of the assignments were accessible via a Word document at the learning platform to which the class was linked. The students’ assignments were formulated as questions as follows:

*‘World trade patterns’* - *assignments* (What is the task you are going to solve?)

1. What changes do you see in the trade patterns existing in the world, as to which goods and services are imported and exported, and which countries do the trading?
2. What are the causes and consequences of the patterns and changes over time?

Also, the students had from the start access to an assessment matrix that included three different levels of knowledge requirements that were specified by three different grade levels, E, C, and A, according to the curriculum.

Each lesson started with a brief review by the teacher, who discussed some phenomena regarding trading patterns with the help of different indicators, demonstrating technical features offered by the tool or explaining the student task more thoroughly. The also gave examples of how to arrange and highlight visual information. In summary the students were asked to study global trade patterns, identify possible relationships between different factors, and then present and share their insights and conclusions through an oral and visual presentation using associated visualizations that supported their reasoning. On the sixth occasion, the students were supposed to present/share their knowledge and conclusions they had gained in relation to the assignments. The written instruction for the examination was presented as:

*Examination* (How should you display the knowledge you have acquired and the conclusions you have drawn?)

3. Make an individual ‘digital presentation’.
4. Report your knowledge and conclusions based on the questions.
5. Give an oral presentation of your results and share them using a visualized presentation.

The students’ speeches at the examination lesson lasted from 1 to 16 min, the student stood in front of the classroom, used their ‘digital presentation’, and shared his/her knowledge with the teacher and a smaller group of students from the class. Each student’s presentation was recorded by the teacher via a screen-recording tool. This documentation was to be used by the teacher for grading the students.

### Video captures

Methods of documentation and analysis that advance knowledge about relationships during activities are of particular interest when studying how things are brought together and function during practices of communication (Heath, Hindmarsh & Luff, 2010). Therefore, video recordings were made to facilitate thorough documentation of the emerging practices in the classrooms when the students conducted their presentations which they had created based on their own visual discoveries. The presentations, at the examination lesson, were recorded by a video camera placed in the back of the classroom. The captures included the faces, voices and gestures of the students as well as the articulations from their digital presentation on the whiteboard. The 30 captured speeches/reports were in average 4–7 min.

### Analyses

Our unpacking of the different phases in the analytical process are the outcomes of rigours work and focus on details, but at the same time the process is a product of choices (Ashmore & Reed, 2000). As part of the analysis process, the video captures were first viewed by one of the researchers. Parts of the recordings identified as crucial to the aim were then viewed several times by both researchers and transcribed. A certain interest was focused on multitude aspects that are involved during the presentations (see Fig. 1). A multitude that can be a challenge both to teachers and us as researchers. Hence, we have tried to engage with the data by attuning to the shifting connectivity of persons and objects (MacLure, 2013), in an open-ended practice of sense-making (Massumi, 2002). In this process, we have used the notion of semiotic assemblage to analyse the interactions between linguistics, visual properties, verbal speech, bodies, and other spatial resources that may emerge in the studied communicational space of the classroom (Deleuze & Guattari 1987). The concept spatial repertoire is further applied to understand the emergent and interactant affordances of such ‘communicational spaces’ (Pennycook, 2018).

In order to both capture and illustrate the richness in the data we have made two crucial choices in *reporting* our results. Firstly, we begin the report of our results with a depiction divided in to visual, bodily and verbal dimensions of communication (see previous research and Fig. 1). Secondly, we base the illumination on empirical examples from one significant event in one of the presentations. The latter is to facilitate the understanding of how these aspects coexist in the selected event. The selected event, that is short (in time), was chosen because of its expressive, transformative connectivity and enactments, which contributes to demonstrate the results in relation to the wider scope of the analysis. Other presentations and episodes could have been part in the account of our results. But since this is a rather novel attempt to grasp the comprehensiveness and complexity, in new circumstances for schools when students present their insights in multimodal ways after working with interactive statistical visualizations, that would have been risked obscuring the main points for both researchers and readers. On the other hand, there is nothing in the other presentations that changes the implications in our results. In the described consideration above we take Ochs (1999; [1979]) and Ashmore and Reed’s (2000) stance by applying a critical reflexive standpoint in relation to the analytical object. Thereby, we are aware of and have considered the possible changes during the ‘stages’ from *event* to *tape* and on to *transcription* and *text*.

The event included in the result section is presented in the form of descriptive verbalisations and selected pictures of a semiotic assemblage in the studied classroom.

## Results

The analysis shows how various dimensions of multimodal communication compile, amass, and serve during practices of communicating a message of visual discoveries in a classroom. In the following a specific detailed *event* from the classroom, named “In short”, is presented as a whole to give empirical evidence to the analysis. Thereafter, the analysis follows.

### A communicational space of spatial and temporal entanglements

In the 22-second-long event, Helene, one of the students, is in the middle of her presentation addressing the other students and the teacher focusing on the insights she has gained with the Vislets in Statistics eXplorer according to the assignment about ‘world trade patterns’.

*The event “In short”.*

A computer is placed at a small, high table which stands beside a brightly lit smartboard at the front of the classroom. Helene stands firmly behind the computer with her hands at one moment on the desk and then gesturing in the air as she talks. Facing her classmates and the teacher, she is trying to explain the concept of imports.

On the smartboard, various ‘items’, including a timeline, arrows, text, numbers, and forms as circles and rectangles, appear on the PowerPoint slide. Canada is used as an example, and the timeline shows Canada’s imports over time, from 1980 to 2012. The timeline is positioned at the bottom of the slide, reaching from the far-left-hand side across the slide to the right-hand side. Four black arrows point towards different years on the timeline, highlighting historical events. Specific texts on the slide are enclosed a rectangle or a circle. One arrow points to the text in such framing (the circle). For example, 1988 and what happened then (a commercial agreement between Canada and the USA). In the upper right-hand corner, there is a textbox forecasting that Canada’s imports will continue to grow (see

**Fig. 4:**
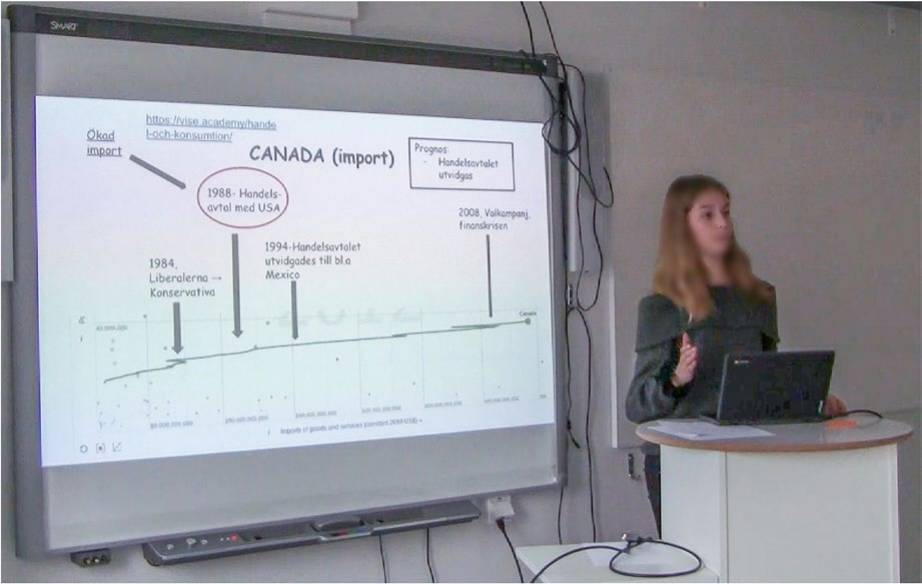
Canada’s import illustrated at the slide in the digital presentation and hand movements accompanies the conclusions.

Fig. 4). Meanwhile, facing the class with her gaze Helene summarizes with a verbal utterance at the same time as she underlines the conclusion with a hand gesture. The gesture includes a vertical hand movement. The hand moves repeatedly and promptly up and down in a small distinct operation (see Fig. 4).

When summarizing, Helene’s tone is engaged but relaxed and in a clear and well-articulated voice she says:

- So, in short, what happened was, the imports increased after the trade and commercial agreement with the USA, in 1988.

At the same time, Helene breaks eye contact with the audience as she turns her head and gazes towards the smartboard. Simultaneously, Helene’s arm moves from the table and performs a large gesture towards the red circle highlighting 1988 on the smartboard (see

**Fig. 5:**
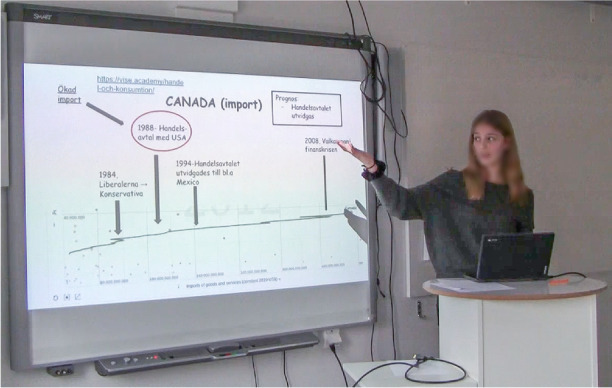
The attention is directed towards the smartboard by non-verbal gestures.

Fig. 5). The gazes of the audience move, from first mostly being directed towards Helene’s face to instead following the hand gesture, which directs the gazes towards the smartboard (see Fig. 5).

Almost concurrently with the oral statement and the gesture, Helene glances at the rectangle in the upper right-hand corner (see

**Fig. 6:**
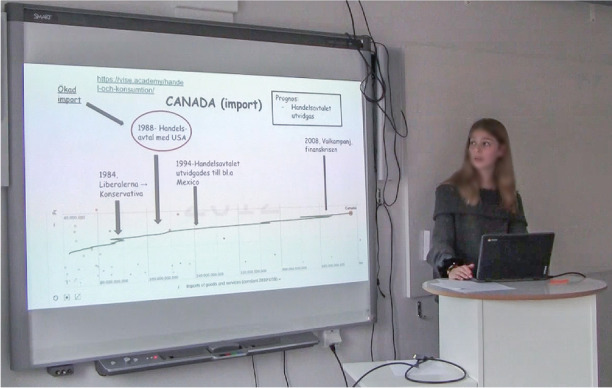
Oral statement, gesture and glance reinforce each other.

Fig. 6).

Thereupon, she makes a forecast:

- With this background, I think it’s possible that this trade and commercial agreement will increase even more …

This utterance includes a very short oral pause after “I think …” and slight emphasis on the word “increase …” . She elaborates further on this conclusion and continues steadily with both a congenial speech rate and good speech fluency. A small gesture is made towards the timeline and the highlighted year 1994. Concurrently, she states that such an expansion happened during that year. Again, her gaze turns towards the class, and simultaneously she stretches out both arms and says:

- “There’s no reason why this would not happen”, and in tandem she shakes her head (see

**Fig. 7:**
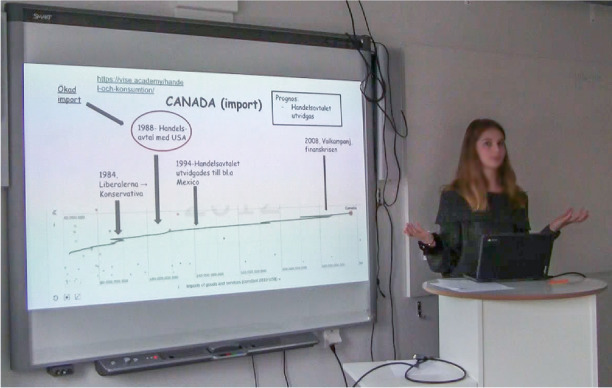
Simultaneous utterance and movement with the head emphasize the student’s conclusion.

Fig. 7).

In this classroom event it is evident, in similarity to other students’ presentations in this study, how various semiotic resources are distributed within the communicative space. They all play roles in the action; they take on meaning and are part of communicative routines. The communication in the classroom is distributed in complex, intense and entangled socio-material interaction. It is distributed by and among visual, bodily, and verbal properties (see below) which may interpellate the listener. The student’s presentation is brought into ‘being’ by these spatial repertoires, they give identity to the message - the knowledge gained by Helene, the student.

In order to further analyse how various modes (visual, bodily and verbal) are brought together and function when students’ findings from visualized statistics are presented in a social science classroom – the entwined relationships among the range of forms of sign processes – it is analytically necessary to disassemble the semiotic assemblage. This means, the interactant affordances, the spatial repertoires that are parts of the communicative routines distributing the semiotic assemblage. Therefore, by picking out visual, bodily, and verbal dimensions of communication, one at a time, we illustrate the characteristics of these spatial repertoires and their semiotic roles (cf. Fig. 1) in three subsections. In the final part of the result section, the semiotic assemblage will be ‘put together’ again to give evidence of the characteristics of the communication – the constructed space of the message.

#### Visual communication

As described, when the presentation – Canada’s imports – is communicated in the classroom, a range of semiotic resources is distributed as an emerging communicative space. Several spatial repertoires circulate in an effort to achieve shared insights with the teacher and the other students. The digital presentation slide function through its visual dimensions by displaying some of the visual discoveries about Canada’s importing of goods made by the student (cf. Fig. 4). Thus, it supports the processes of sharing the identified knowledge, to convey the student’s message, and to introduce an idea or conclusion. However, the presented visualized insights now take on a different ‘form’ than before because they are generated out of the analysis and mapping of mass data and then visualized again, on the slide, by Helene, the student. In the presentations design space considerations, although not always as careful as in this event, are carefully made with the purpose of pursuing captured findings, eventually to make awareness and inferences easier, and to gain shared insights with the teacher and the other students. To facilitate an understanding of Helene’s discoveries, different ‘items’ such as a timeline, arrows, text, numbers, circles and rectangles, link ‘the message’ of these visual properties. So, there are choices made based on visual narrative tactics and design space considerations. These choices support the communication of the messages and are considered to help the introduction of the student’s novel insights and make these insights more permanent. By placing the diagram, which is a screenshot from Statistics eXplorer, showing Canada’s imports over time, at the bottom of the slide, reaching from the far left-hand side to the right-hand side, we perceive this ‘item’ to work as a balancing base for the image. This emphasizes the information value as the created zone also indicates a reading of this slide from left to right, because the timeline starts in the bottom left-hand corner and continues to the right. To highlight and connect elements of the slide, the student has added four black arrows pointing towards different time periods and historical events on the timeline. This framing underlines and explains changes in development of Canada’s imports, such as important elections, a trade treaty, and a financial crisis. These events have been discovered by the student in other sources, in addition to the statistics offered by the VA (i.e., webpages).

Specific words or text on the slide are also enclosed in different shapes, such as a rectangle or a circle, or arrows are put close the text. This adds salience to the text as it becomes highlighted in this way. For example, the circle around the year 1988 and the text about what happened then (the commercial agreement between Canada and the USA) gives a specific emphasis to this phenomenon, which we comprehend as having a central meaningful function in the knowledge-sharing message. By also using a *red* circle around it, the appearance is emphasized even more. The red colour makes the item ‘stand out’ on the slide. The text box (in black) in the upper right-hand corner concludes the visual message with a forecast that Canada’s imports will continue to grow.

To summarize, the slide provides a combinable understanding that reframes/presents relevant concepts and a narrative of trade patterns in a visual, playful way. It offers a set of visual narrative tactics used to persuade the teacher and fellow students that this knowledge is valid and an authentic way to view the issue (cf. Fig. 1).

#### Bodily communication

Alongside the visual communication, both bodily and verbal dimensions are involved in the presentation which exemplifies important aspects of the communicative routines at play. The wide-angle recording captured how Helene strives to establish eye contact with the audience through mostly looking at them with a rather intense or firm gaze. There are also examples of distinct spatial behaviours. For example, when Helene’s head and gaze are directed towards the smartboard in a way that also invites the other students and the teacher to follow her motions and look at the screen (cf. Fig. 6). This spatial behaviour is further enhanced during the episode when her right hand moves from the table and forms a large gesture towards the smartboard. During the presentation, a variety of different reinforcing non-verbal gestures occur in this way (cf. Fig. 5). A repeated posture she does during the presentation is to lean her body forward or backward to what is interpreted as either exaggerate something or move forward to the next item in the presentation. During other parts there are examples of a mixture of gestures, body postures and voice inflection, accentuation, articulation, breaks, intonation or volume pitch. For example, the presentation begins with Helen firmly pulling up the sleeves of her sweater and at the same time saying: “Now we start”. This is uttered with a rather strong intonation which works to give a bodily auditory impression. Other examples of this are, when summarizing an argument. Then Helen sometimes uses body movements to emphasise and her tone changes; it is still relaxed but with more engagement and well-articulated. This is illustrated in Fig. 7, when she stretches out both arms and, at the same time, shakes her head and with her articulation and voice pitch emphasizes a chain of arguments.

#### Verbal communication

As already stated, the auditory impression is quite clear when Helene pulls up her sleeves and simultaneously says: “Now we start”. Nevertheless, this is also an example of the importance of verbal language and how verbal and bodily attributes are inseparable parts of spatial repertoires. It signals the start of the presentation and attracts attention. On a recurring basis, Helene also stops and explain the meaning of different concepts. When the picture in Fig. 4 appears on the screen, for example, she verbally defines ‘import’. By dint of personal addresses, such as “what we see here is”, and predictions of future developments, the verbal utterances complement other factors active during the presentation.

So, Helene verbally guides her audience in different ways, for example, by summarizing the content of different parts of her presentation. Sometimes this is done in the form of her own conclusions. But she also supports the listener through comments about her presentation. She begins by stating what the presentation will be about. Another example is when she says: “I’ve now talked about exports and will now continue about imports, with Canada as an example.” This is an example of how the speech in this presentation is organized. Even though, as described in previous research, nuances in verbal communication are not as obvious as visual and bodily communication, in this event verbal communication, such as verbal utterances and the organization of the presentation, are crucial elements of the spatial repertoires at play.

### ‘The message’ produced by synchronic spatial repertoires

The purpose of the presentation in the event is to communicate insights and knowledge to a group of classmates and the teacher. In this case, it has been demonstrated how the spatial distribution, the social practices, and material embodiment emerge in such a way that the message become acceptable, readable, and combinable and thereby establishes an authenticity of the shared knowledge (see Fig. 1). Alongside verbal language, diverse tools are ‘utilized’ during the presentation as an extended visual narrative, facial expressions, gazes, gestures, bodily orientation, spatial indications, movement, images, a variety of material objects, etc. These spatial repertoires, draws on different semiotic modes, but the modalities are integrated in such a way that they provide a ‘cohesive’ element in/of the communication. The interactant affordances of the spatial repertoires, are thus a part of the message, the communicated presentation in the classroom. The fusing of them contributes to expressing the ideational and what are to become inter-bodily components of the message (the gained knowledge that is to be shared), where communication occurs by the attuning of (socio and material) bodies (Pennycook, 2018). So, the ‘message’ presented is created by a combination of spatial repertoires that communicate meaning with rhetorical force.

After all, the repertoires with all the inherent qualities contribute to the communicative space, they all constitute the processes of delivering a ‘reliable’ (acceptable, readable, combinable) message. Generally, it is difficult to evaluate whether the content of a multimodal message is judged as authentic by the receivers (see Fig. 1). Since this presentation is conducted in a school, the final judgement and assessment is left to the teacher. However, in this study, the focus is on the students performing their presentations, and not on the dialogue between the participants or how the teacher conducts the grading process.

### ‘The message’ as distributed language: A semiotic assemblage

By now finally” putting together” the semiotic assemblage in the 22-second-long event it become evident how multifaceted and complex the undertaking of distributing a multimodal message is – how ‘things’ may assemble and operate in students’ presentations of their insights, their ‘proof” of gained knowledge. The assemblage demonstrates a complexity of the vibrant, intermingling exchanges occurring. All the different parts, the spatial repertoires, the communicative routines in the presentation configure and disseminate in and through the emerging semiosis. They are part of the action; they are part of the message about Canada’s import. ‘The message’ works through a communicative space of spatial and temporal entanglements, a distributed language. This view of language as distributed by the intermingling of communicational spaces, as analysed above, may challenges the idea of language as an internalized system or individual competence (Pennycook, 2018). However, the various individual presentations were communicative processes in which the message, and the language, was embodied, embedded, and distributed across people (in the classroom), across places (the classroom – this text) and time (the present/past/future). The analysis of the empirical material strengthens an understanding of communication as being, not a process of choosing among a predetermined set of options, but a set of open-ended opportunities. This complexity of communication, the things that come together in particular semiotic assemblages, at particular moments of time and space needs to be addressed in classrooms.

## Discussion

Using Latour’s (1990) accounts of how images and inscriptions provide advantages in rhetorical dialogues as one starting point, this study has demonstrated that this can also be applied in an analysis of what happens during a student’s presentation in school. The study has explored modes that may be flexible and modifiable in aiding reasoning, reflection, and the linking of items in elementary students’ presentations of their insights in relation to social-science content. Of course, these presentations are affected by the intervention, the role of the teacher, the arrangements of the information in the Vislet in the VA-application, and etcetera. As in all teaching the benefit from these kinds of aspects affect the students in various ways. However, this study does not focus on the process of how the students came up with their insight’s, instead its concerns is directed towards the presentation of the students’ comprehensions, how the message of their knowledge is communicated when they are encouraged by their teachers to present their visual discoveries in various ‘novel’ ways as a final stage in the knowledge-building process. Evident is how the communication of such “messages”, formed by various modes (visual, bodily and verbal) brought together, emerge as spatial distribution, social practices, and material embodiment, rather than being only a matter of the student’s individual competence as a sociolinguistic actor.

Therefore, the study underlines the importance of looking at the language of such messages and knowledge sharing as not so much a linguistic system and choices made by the student, but as a greater totality of interacting objects, places, and various forms of semiosis. When semiotic assemblage is successfully used as a lens while attempting to understand how students can work with data visualizations, complexity and open-ended opportunities when sharing their knowledge are one result. In turn, this might influence not only previous understandings of communication but also the terms for assessments in educational practices, as well as how knowledge will be understood and assessed in school. It is not enough for teachers to use a pre-fabric assessment matrix as was the case in the studied classrooms.

For a long time, schools have been moving away from the examination as only a written text in which the student is supposed to repeat content from a given source. If this ever was truly the situation. Today, students can gather information from multiple sources, including advanced VA combined with various webpages. Previous research has shown that students’ findings and insights barely can be transformed into written text because the data visualizations contain so much condensed information. Instead, as is shown in this study, oral presentations combined with visuals and texts on slides can serve as a more flexible and modifiable mode. In any case, such multimodal presentations, analysed as semiotic assemblages, confront teachers with the need to assess not only dimensions of relevant facts but also how the presentations are conducted. This conclusion is obviously also relevant when students only hand in a written text – but there are different and more aspects when considering a presentation combining visual, bodily, and verbal dimensions of communication addressing a group of other students and the teacher. It has been shown that spatial repertoires, visual, bodily, and verbal, act, promote, and form the presentation. In this respect, ‘knowledge’, already a multifaced concept, is broadened even more and will have to include, for example, visual competences, the use of gestures, and intonation – how ‘things’ assemble, operate, and function. If this is correct, then teachers and schools will be required to consider various dimensions of communication – the entanglement of semiotic assemblages – when assessing student achievement. It is not only a question of incorporating more aspects when judging student performance, from a longer perspective, this will also affect the view of what kinds of knowledge and skills schools should strive towards. This implicates the importance to understand and train students in communication skills applicable for a technified, contemporary society. Both society and schools have gone through such changes previously, but nevertheless it is a transformative process in which the outcome is hard to foresee and thus stressful. For example, a conflicting situation is now emerging since audio, speech, and discussions are not easily grasped and are seldom taken into account as evidence of knowledge.

Therefore, VA, as implemented data visualizations, has the potential to, in some extent, unload the burden of teachers to possess huge amounts of information relevant to their students. Instead, teachers can support their students in seeking, discussing, and integrating information in collaborative knowledge-building processes, but they may simultaneously be forced to try out diversified ways of viewing knowledge and assessment (Baldwin, 2015).

The study has been conducted at an early stage of using VA in schools as part of a larger project. In this study students have been allowed to do their own discoveries and using VA for presentations. At the same time the ambition has been to find out how modes like visual, bodily and verbal were brought together and function during the students’ knowledge sharing. This is a novel area for inquiry and since there are no established modes for presenting elaborated results on these complex matters, we have chosen to account for our results based on empirical examples from a short sequence in one of the presentations. Although our analysis is based on all presentations. To choose one empirical event for illuminating the results is foremost motivated to make them easier to embrace and comprehend. We expect and hope to be able to contribute to methods both for analysis and accounts, bringing insights developed for such multifaceted, multimodal activities. A development that has occurred over time in other novel fields (Godhe et al., 2021).

Further empirical work and analyses are to come, since we are convinced that VA and other similar AI tools will soon be situated in schools to a degree that is hard to grasp today. Precisely because of that magnitude, it is vital to conduct exploratory and critical studies of multifaceted processes of communication concerning students’ knowledge sharing as the final stage in the ‘cognitive’ process in schools.

## Footnotes

1 More information of Statistics eXplorer is available at National Center for Visual Analytics (2017)

2 Methods and procedures for the collection, processing, storage, and presentation of official statistical data should consider scientific principles and professional ethics (United Nations Statistics, 2014)
